# Clinical Isolates of Antimicrobial-Resistant Enterobacter Species Can Persist in Human Macrophages Without Replication and Overt Cellular Cytotoxicity

**DOI:** 10.1093/infdis/jiaf099

**Published:** 2025-03-03

**Authors:** Georgiana Parau, Hannah J Parks, Amy J G Anderson, Fabiana Bisaro, Inmaculada García-Romero, Michael C Gilmore, Samuel O Korankye, Helina Marshall, Miguel A Valvano

**Affiliations:** Infection Biology Group, Wellcome-Wolfson Institute for Experimental Medicine, Queen's University Belfast, Belfast, United Kingdom; Infection Biology Group, Wellcome-Wolfson Institute for Experimental Medicine, Queen's University Belfast, Belfast, United Kingdom; Infection Biology Group, Wellcome-Wolfson Institute for Experimental Medicine, Queen's University Belfast, Belfast, United Kingdom; Infection Biology Group, Wellcome-Wolfson Institute for Experimental Medicine, Queen's University Belfast, Belfast, United Kingdom; Infection Biology Group, Wellcome-Wolfson Institute for Experimental Medicine, Queen's University Belfast, Belfast, United Kingdom; Centro Andaluz de Biología del Desarrollo, Consejo Superior de Investigaciones Científicas-Universidad Pablo de Olavide, Sevilla, Spain; Infection Biology Group, Wellcome-Wolfson Institute for Experimental Medicine, Queen's University Belfast, Belfast, United Kingdom; Infection Biology Group, Wellcome-Wolfson Institute for Experimental Medicine, Queen's University Belfast, Belfast, United Kingdom; Strathclyde Institute of Pharmacy and Biomedical Sciences, University of Strathclyde, Glasgow, United Kingdom; Infection Biology Group, Wellcome-Wolfson Institute for Experimental Medicine, Queen's University Belfast, Belfast, United Kingdom

**Keywords:** ESKAPE, pyroptosis, antimicrobial resistance, intracellular survival, cytotoxicity

## Abstract

**Background:**

*Enterobacter* species are opportunistic, multidrug resistant gram-negative bacteria associated with morbidity and mortality worldwide. Because very little is known about the infection biology of *Enterobacter* spp, we investigated the intracellular trafficking of a subset of *Enterobacter* clinical isolates, including colistin-resistant strains, within human macrophages and determined the macrophage response to the intracellular infection.

**Methods:**

Phagocytosis of 11 clinical isolates representing *Enterobacter cloacae, Enterobacter bugandensis, Enterobacter kobei, Enterobacter xiangfangensis, Enterobacter roggenkampii, Enterobacter hoffmannii,* and *Enterobacter ludwigii* was investigated in primary human macrophages. Intracellular bacterial trafficking was followed by confocal fluorescence microscopy, intracellular bacterial replication was assessed by bacterial enumeration, and a fluorescence dilution approach was used to follow bacterial cell division over time. Macrophage cell cytotoxicity was investigated by quantifying the release of lactate dehydrogenase during infection and by determining cleavage of the proinflammatory markers caspase-1, gasdermin D, and prointerleukin-1β.

**Results:**

*Enterobacter* isolates did not replicate in human macrophages, exhibiting long-term survival (up to 44 hours) within a modified late phagolysosome compartment. Survival did not correlate with colistin resistance, lipopolysaccharide modifications, or bacterial pathogenicity in the *Galleria mellonella* infection model. Intracellular bacteria induced low levels of macrophage cytotoxicity that correlated with absence of cleavage of proinflammatory markers in infected macrophages.

**Conclusions:**

*Enterobacter* spp clinical isolates can persist without replication inside human macrophages with minimal effects on cell integrity and inflammation. These observations could have implications for clinical outcome of patients that cannot readily clear *Enterobacter* infections, which can potentially lead to prolonged intracellular survival and infection relapse.

## Teaser

This study underscores the capacity of antibiotic-resistant *Enterobacter* clinical isolates to persist in macrophages without significantly alerting the innate immune system, which may further complicate the treatment of *Enterobacter* infections leading to treatment failures and recurrent infections.

*Enterobacter* species are facultative, anaerobic, gram-negative bacteria of the Enterobacteriaceae family. The taxonomy of *Enterobacter* is evolving rapidly, resulting in the designation of numerous new species [1]. Many *Enterobacter* species [2, 3] are present in the human gut microbiota, but also can cause bacteremia, neonatal sepsis [4–6], and infections in multiple sites [2, 7, 8]. They are also found in aquatic and terrestrial environments, and some species can be symbiotic or pathogenic for plants and insects [9–12]. Infections are difficult to treat due to multidrug antibiotic resistance including resistance to carbapenems and last-resort antibiotics such as polymyxins [13]; for this reason, *Enterobacter* species are included in the World Health Organization’s high priority list of global threat pathogens [14] for which new antibiotics are urgently needed.

Despite abundant information on antibiotic resistance in *Enterobacter* species, much less is known about their infection biology, particularly the ability of *Enterobacter* species to interact with innate immune cells such as macrophages and neutrophils. We searched PubMed with the terms “*Enterobacter*” AND “macrophage,” “*Enterobacter*” AND “monocytes,” and “*Enterobacter*” AND “intracellular” for original articles published in English up to 9 July 2024. The search excluded terms “*sakazakii*” and “*aerogenes*” because *Enterobacter aerogenes* and *Enterobacter sakazakii* have been moved to the genus *Klebsiella* and *Cronobacter*, respectively. Of the identified 55, 15, and 181 studies, respectively, only 1 reported direct testing of *Enterobacter cloacae* phagocytosis [15]. A few studies reported survival of *E. cloacae* and *Enterobacter hormaechei* in human monocytic macrophage cell lines [16–18]. *E. cloacae* was also reported to form intracellular bacterial communities in uroepithelial cells [19], and several *Enterobacter* species can induce apoptosis in human epithelial cells [20–22]. Expanding the search using “Enterobacter” AND “infection” did not afford any additional studies. We concluded that the mechanisms by which *Enterobacter* clinical isolates survive intracellularly remain unexplored. In this study, we have investigated the intracellular trafficking of several antibiotic-resistant *Enterobacter* clinical isolates within human macrophages and determined the macrophage cellular response to the infection using two prototypic species: *E. cloacae* American Type Culture Collection (ATCC)13047 and *Enterobacter bugandensis* 104107. Our data demonstrate that *Enterobacter* strains from several different species can survive in human macrophages with no replication. Bacterial survival occurs in a modified late phagolysosome compartment with reduced acidity and does not induce overt cytotoxicity. These findings underscore the capacity of *Enterobacter* clinical isolates, traditionally viewed as extracellular bacteria, to establish a protective niche in macrophages and possibly delay detection by the innate immune system. This may further complicate the treatment of infections in susceptible populations.

## METHODS

### Bacterial Strains and Culture Conditions

The *Enterobacter* clinical isolates were collected from bloodstream and lower respiratory infections through the British Society of Antimicrobial Chemotherapy surveillance programs [23] and investigated in a case-control comparison of colistin-resistant and -susceptible isolates representative of various *Enterobacter* species [24]. The 11 clinical isolates used in this study were *E. bugandensis* (2 isolates), *Enterobacter kobei* (2 isolates), *Enterobacter xiangfangensis* (2 isolates), *Enterobacter roggenkampii* (1 isolate), *Enterobacter hoffmannii* (1 isolate), *Enterobacter ludwigii* (2 isolates), and the *E. cloacae* type strain ATCC13047. Growth conditions, antibiotic susceptibility and taxonomy of these isolates are described in the Supplementary Methods.

### *Galleria mellonella* Infection

*Galleria mellonella* larvae (UK Waxworms, ltd), a widely used surrogate infection model [25], was employed to assess the pathogenic potential of the *Enterobacter* isolates, as described in the Supplementary Methods.

### Lipid A Mass Spectrometry

Matrix-assisted laser desorption/ionization-time of flight (MALDI-TOF) mass spectrometry (MS) was used to characterize lipid A modifications in the lipopolysaccharide (LPS) of strains, with and without challenge with polymyxin B (a colistin analogue), as described in more detail in the Supplementary Methods.

### Cell Culture, Macrophage Infection, and Confocal Imaging

THP-1 monocytes (ATCC TIB-202) were cultured in Roswell Park Memorial Institute (RPMI) 1640 medium (Thermo Fisher Scientific) plus 10% fetal bovine serum (Thermo Fisher Scientific), seeded at a density of 2×10^5^ cells/mL, and differentiated into macrophages with 80 ng/mL phorbol 12-myristate 13-acetate (Sigma-Aldrich) for 3 days. Human monocyte-derived macrophages (HMDMs) were isolated from buffy coats obtained from the Northern Ireland Blood Transfusion Service (project reference number 2019/09). Ethical approval for the use of HMDMs isolated from buffy coats was obtained from the Ethics Committee of the Faculty of Medicine, Health and Life Sciences, Queen's University Belfast (reference MLHS 19_22).

THP-1 and HMDM cells were infected with the clinical isolates, using various multiplicities of infection (MOI) (indicated in the figure legends). After 30 minutes, infected macrophages were treated for 30 minutes with 100 μg/mL kanamycin to kill extracellular bacteria and then incubated with 50 μg/mL kanamycin for the remaining duration of the experiments. Macrophages were either lysed to enumerate bacteria or processed for imaging using live and immunofluorescence staining microscopy, as applicable. Additional details are provided in the Supplementary Methods.

### Cytotoxicity Assay

The cytotoxicity of the bacterial infections in macrophages was investigated using the Roche lactate dehydrogenase assay kit with a minimum of 2 technical repeats per experiment and up to 6 biological repeats, as described in the Supplementary Methods.

### Immunoblotting

Experiments were performed at 5 hours postinfection (hpi). Positive controls included LPS-treated (1 μg/mL) macrophages with and without the addition of nigericin (20 µM) to induce pyroptosis and inflammasome priming, respectively. The priming effect of heat-killed bacteria with and without nigericin was also investigated. Blots were incubated with primary rabbit antibodies to detect cleavage of caspase-1, gasdermin D (GSDMD), and interleukin-1β (IL-1β), and subsequent incubation with the secondary goat anti-rabbit IRDye 800 cW (LI-COR) antibody. Membranes were imaged using an LI-COR Odyssey scanner. Additional details are provided in the Supplementary Methods.

### Statistical Analysis

Because colony-forming units (CFU) do not follow a normal distribution, we analyzed the natural log-transformed values using geometric means. For comparison of 2 groups in the microscopy analysis of infected tissue sections that followed a normal distribution, we used a *t* test. For comparison of multiple groups, a one-way ANOVA was done, with Tukey correction for multiple comparisons. *P* values were considered significant if they were less than .05.

## RESULTS

### *E. bugandensis* Strain 104107 Traffics to Late Phagolysosome Compartment in Human Macrophages

**Figure 1.**
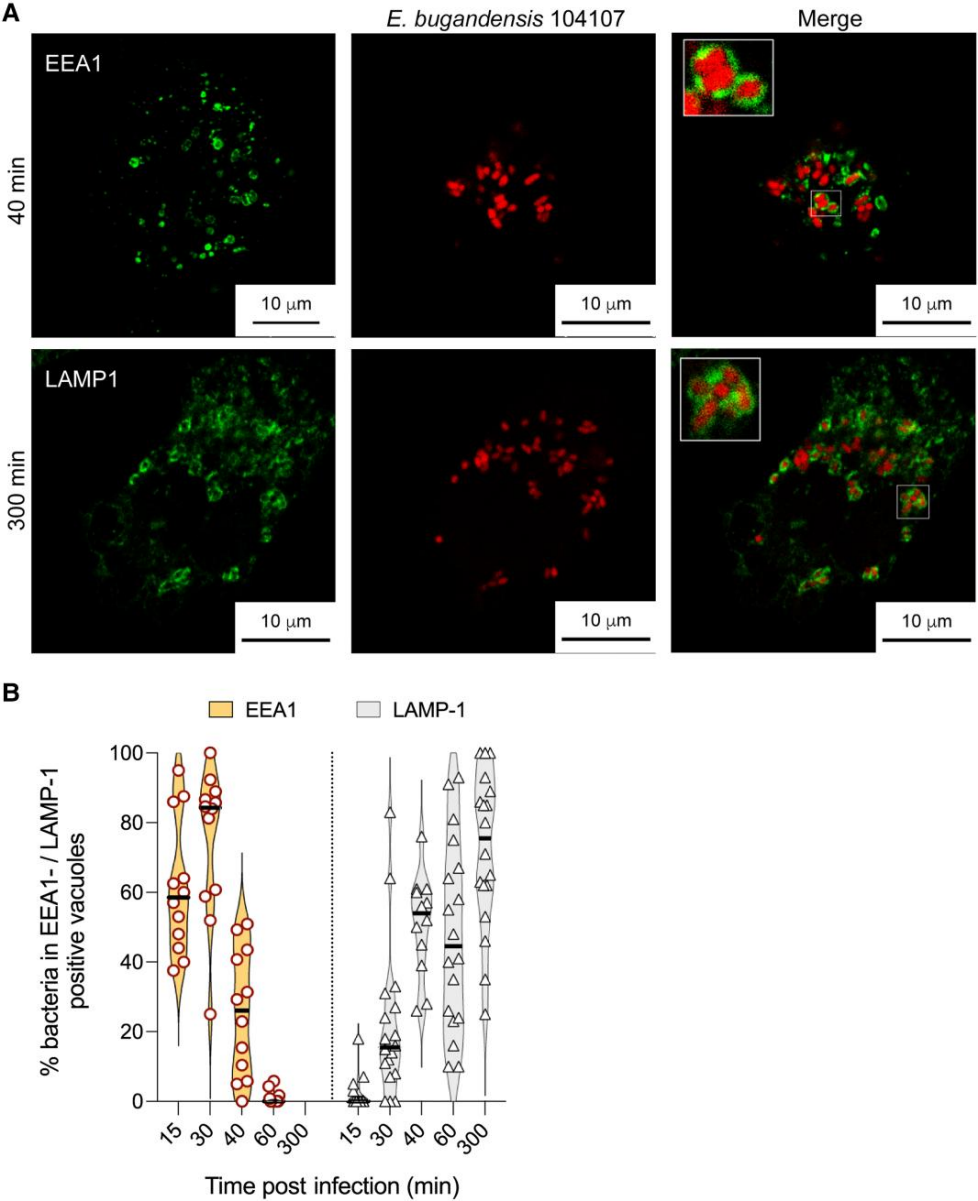
Intracellular trafficking of *Enterobacter bugandensis* 104107 from early to late phagolysosomes in THP-1 macrophages by confocal immunofluorescence microscopy. *A*, Colocalization of live *E. bugandensis* 104107 in membrane vacuoles labelled for the early phagosomal marker EEA1 at 40 minutes postinfection, and vacuoles labelled for the late phagolysosome marker LAMP1 at 300 minutes postinfection (additional images showing all time points postinfection are in Supplementary Figures 2–4). All images were taken with ×63 magnification on a Leica SP8 confocal microscope. MOI = 120. *B*, The percent of intracellular bacteria in EEA1- and LAMP1-positive vacuoles over time. Data were obtained by counting the number of vacuoles with bacteria and the corresponding vacuolar markers in at least 40 macrophages per time point over 3 biological replicates. Horizontal lines indicate means. Abbreviations: EEA1, early endosomal antigen 1; LAMP1, lysosome-associate membrane protein-1; MOI, multiplicity of infection.

Initial experiments to investigate the behavior of clinical *Enterobacter* isolates in human macrophages employed E. bugandensis 104107, a colistin-resistant isolate obtained from respiratory secretions [24, 26]. THP-1 macrophages were infected with 104107 expressing the mCherry red-fluorescent protein. Confocal fluorescence microscopy at early times (15 to 60 minutes) postinfection revealed that the bacteria trafficked into *Enterobacter-*containing vacuoles (ECVs) colocalizing with early endosomal antigen 1 (EEA1), an early phagosomal marker (Figure 1*A* and Supplementary Figure 1). The percentage of ECVs-EEA1 colocalization decreased from 70% at 15 and 30 minutes postinfection to less than 10% at 60 minutes (Figure 1*B*). Transient EEA1 recruitment into phagosomes [27] suggested that intracellular bacteria do not interfere with the initial maturation of ECVs. We next investigated whether ECVs recruit the late marker lysosome-associate membrane protein-1 (LAMP1). Contrary to the results with EEA1, the percentage of ECVs-LAMP1 colocalization increased over time (Figure 1*A* and 1*B*, and Supplementary Figure 2), consistent with traffic of the ECV from an early to a late phagosome. Similar results were obtained in THP-1 cells stably expressing green fluorescent protein (GFP)-tagged LAMP2 (Supplementary Figure 3), another late-phagosome marker; this experiment revealed that intact bacteria could remain in late phagolysosomes for up to 24 hpi.

The observations in THP-1 macrophages were recapitulated in primary HMDMs obtained from buffy coats and differentiated with granulocyte-macrophage colony-stimulating factor (GM-CSF). *E. bugandensis* 104107-infected HMDMs were followed for 3, 5, and 24 hours. The results indicated that intracellular bacteria remained confined to LAMP1-positive vacuoles (Supplementary Figure 4). The infection of HMDMs obtained after stimulation with M-CSF (Supplementary Figure 4), which induces macrophage polarization into anti-inflammatory phenotypes [28], demonstrated that the bacteria also remained within LAMP1 compartments up to 44 hpi. Therefore, we conclude that *E. bugandensis* 104107 can infect primary human macrophages, residing in a late phagosome for a relatively long time.

### Intracellular Bacteria Reside in Vacuoles During Infection and Do Not Traffic Into Autophagosomes

**Figure 2.**
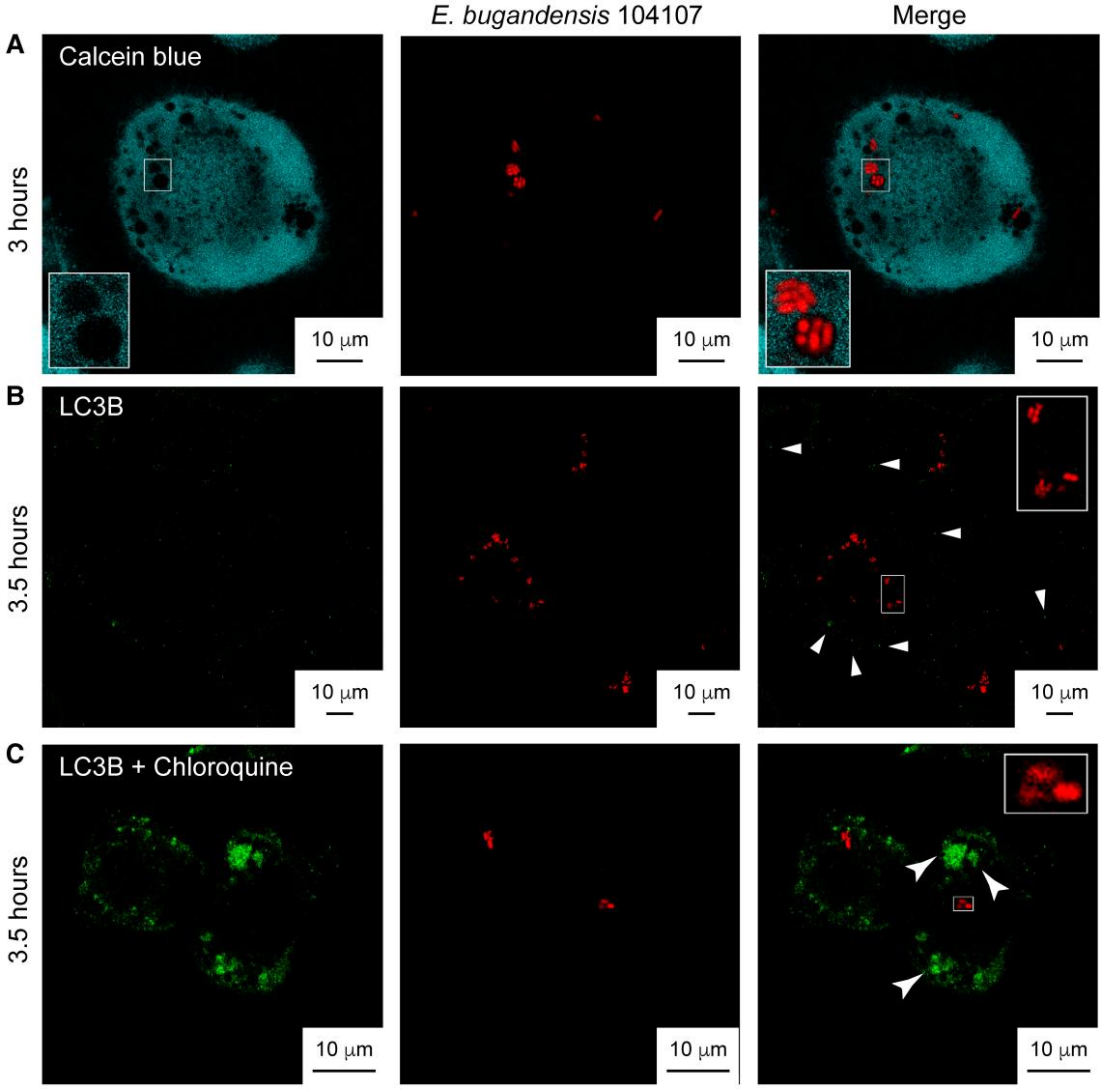
Intracellular *Enterobacter bugandensis* 104107 remain within membrane vacuoles and do not traffic to autophagosomes. *A*, Live cell imaging of THP-1 macrophages infected with *E. bugandensis* 104107 at 3 hours postinfection and stained with calcein blue, which does not enter membrane compartments. Images taken on a Leica Stellaris-5 confocal microscope with ×100 magnification. Multiplicity of infection (MOI) = 15. (Additional images at 7 hours postinfection and a 3-dimensional reconstruction model of an infected calcein blue-stained macrophage cell are shown in Supplementary Figure 5). *B*, Immunofluorescent microscopy images of infected macrophages stained for the autophagy marker LC3B at 3.5 hours postinfection. Arrows in the merged image indicate LC3B puncta that do not colocalize with the intracellular bacteria. *C*, Similar experiment as in (*B*) with macrophages treated with 70 μM chloroquine to inhibit autophagy, which shows accumulation of the LC3B marker in larger clumps (arrows) but without colocalization with the intracellular bacteria. *B* and *C*, Images were taken with ×63 magnification on a Leica SP8 confocal microscope. MOI = 120.

Live microscopy of infected macrophages using the vital dye calcein blue, which becomes membrane-impermeable once inside the cytosolic compartment [29], revealed that bacteria remained within vacuoles (Figure 2*A*). Tridimensional reconstruction of multiple Z-stacked images of single cells confirmed that internalized bacteria were within vacuoles (Supplementary Figure 5).

Because some intracellular bacteria, like *Burkholderia cenocepacia*, acquire autophagy markers early after engulfment by macrophages, as demonstrated by recruitment of the autophagy protein LC3B to the bacteria-containing vacuole [30], we assessed whether LC3B was associated with ECVs. As a control, THP-1 cells were treated with 70 µM chloroquine overnight, which inhibits autophagy by interfering with the fusion of autophagosomes with lysosomes [31]. Only diffuse fluorescent LC3B puncta were observed throughout infection, but these were not in association with fluorescent bacteria, and while autophagosomes were detected in the chloroquine-treated cells as bright fluorescent areas of dense LC3B accumulation, none of these contained bacteria (Figure 2*B* and *C*). In contrast, LC3B was associated with *B. cenocepacia-*containing vacuoles (Supplementary Figure 6). Together, these experiments suggest that ECVs do not traffic into autophagosomes.

### *E. bugandensis* 104107 Resides in a Modified Late Phagolysosome

**Figure 3.**
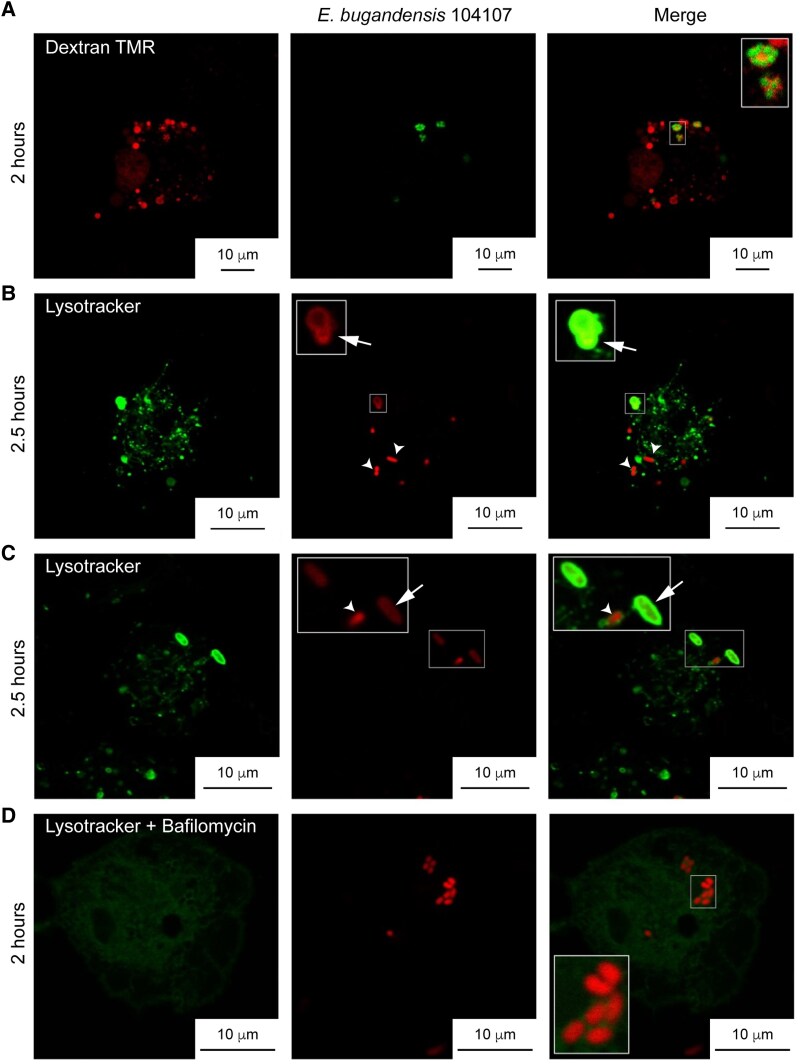
Intracellular *Enterobacter bugandensis* 104107 can traffic to a phagolysosome that delays acidification. *A*, THP-1 macrophages were pretreated before infection with the fluid phase marker dextran-TMR to preload phagolysosomes with red-fluorescent particles. Cells were infected with *E. bugandensis* 104107 containing pLS2 (encoding GFP) and imaged 2 hours postinfection at ×100 magnification on an SP8 confocal microscope. MOI = 50. *B*, THP-1 macrophages infected with *E. bugandensis* 104107 expressing mCherry were treated with the fluid phase marker Lysotracker green DND-26 and imaged at 2.5 hours postinfection. Arrows indicate bright, acidic vacuoles where the entire vacuolar lumen was red-fluorescent. Arrowheads point to intact bacteria that did not colocalize with Lysotracker. *C*, Another field of view from the same experiment as in (*B*) showing weakly fluorescent bacteria that colocalize with Lysotracker-stained vacuoles (arrows), while brightly fluorescent bacteria do not (arrowheads). *D*, Infected THP-1 macrophages treated with bafilomycin to inhibit phagosomal acidification did not show bacterial colocalization with Lysotracker at 2 hours postinfection; all bacteria appear to be intact and display similar levels of fluorescence. *B* and *C*, Live fluorescent images were captured using a Leica Stellaris-5 confocal microscope, × 100 magnification. MOI = 15. Abbreviations: GFP, green fluorescent protein; MOI, multiplicity of infection; TMR, tetramethylrhodamine.

To better define the ECVs, we investigated the 104107 colocalization with dextran-TMR (tetramethylrhodamine), a fluid-phase marker that traffics to lysosomes. THP-1 macrophages prelabelled with dextran were infected with 104107 for 2 hours. Confocal microscopy revealed that bacteria colocalized with dextran-rich compartments (Figure 3*A*) presumed to be lysosomes. One other characteristic of lysosomes is their low pH. We estimated the intraluminal pH of the ECVs using Lysotracker green DND-26 in infected THP-1 cells. Only approximately 25% of intracellular 104107 bacteria were found in bright Lysotracker-positive compartments (Figure 3*B* and 3*C*). Compared to bacteria that did not colocalize with Lysotracker, the bacteria present in the strongly acidic vacuoles appeared weakly labelled and, in some cases, the entire vacuolar lumen was red-fluorescent (Figure 3*B* and 3*C*), suggesting that low pH compromised the bacterial cell envelope integrity [32]. As expected, no colocalization with Lysotracker was observed in cells treated with the vacuolar proton-ATPase inhibitor bafilomycin (Figure 3*D*). Together, these experiments demonstrate that engulfed 104107 mainly traffic into a modified *Enterobacter*-containing late phagosomal compartment that delays acidification.

### *E. bugandensis* 104107 Persist Intracellularly Without Replication

**Figure 4.**
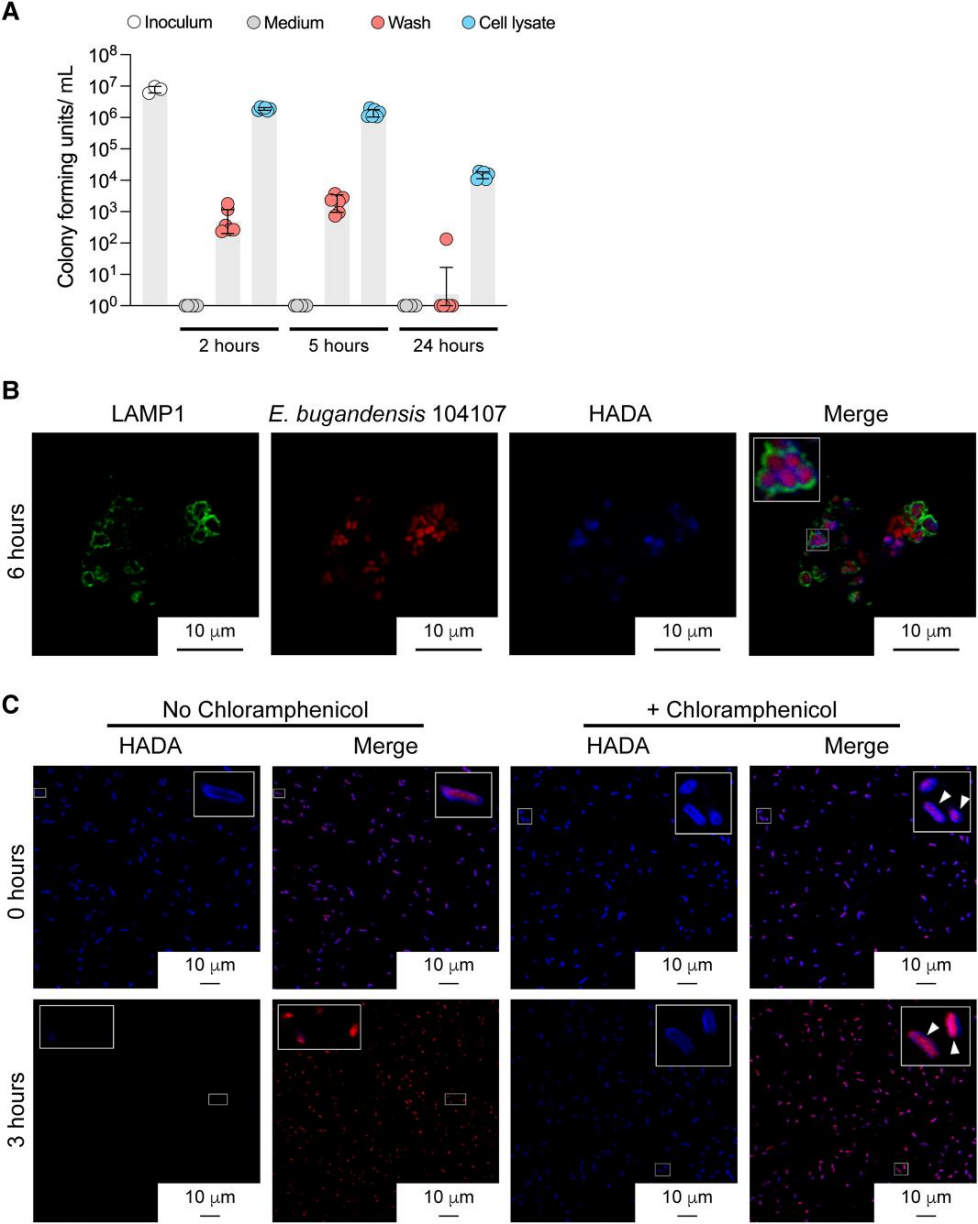
*Enterobacter bugandensis* 104107 bacteria do not replicate in THP-1 macrophages. *A*, Intracellular bacteria enumeration (CFU/mL) at various times postinfection comparing the number of bacteria recovered at each sampling time point with the inoculum at time 0 from the medium (antibiotic control), last wash prior to macrophage lysis (macrophage intactness control), and cell lysate. At 1 hour postinfection, extracellular bacteria were killed by adding 100 μg/mL kanamycin for 30 minutes and then 50 μg/mL remained in the medium for the duration of the experiment. Error bars indicate standard deviation. *B*, Bacteria were pretreated with HADA prior to infection of THP-1 macrophages and imaging by immunofluorescence confocal microscopy at 3 and 6 hours postinfection. Merged image shows HADA-positive bacteria colocalizing with LAMP1. Images were taken with ×100 magnification on a Stellaris-5 confocal microscope. MOI = 15. *C*, Live *E. bugandensis* 104107 were pretreated with HADA, and with or without 50 µg/mL chloramphenicol to prevent replication and imaged live at time 0 (upper row) and 3 hours (lower row). Arrows in the merge images indicate HADA-labelled bacteria at time 0 (upper row), and the absence of HADA labelling after 3-hour incubation at 37°C (lower row). Abbreviations: CFU, colony-forming units; HADA, 7-hydroxycoumarincarbonylamino-D-alanine; LAMP1, lysosome-associate membrane protein-1; MOI, multiplicity of infection.

The bacterial load in infected macrophages was quantified by treating extracellular bacteria with kanamycin throughout the course of infection. While bacteria were not detected in the medium, macrophage cell lysates upon detergent treatment at 2 and 5 hpi revealed a similar bacterial load as the inoculum at time 0 (Figure 4*A*). The lysate at 24 hpi showed a 3-fold reduction in bacterial counts. Low number of bacterial counts was detected in the last wash prior to detergent-mediated cell lysis at all sampling times, which we attributed to a low level of spontaneous macrophage lysis (Figure 4*A*). Overall, the recovery of intracellular bacteria over time at similar loads as the initial inoculum demonstrates that the bacteria remained viable but did not replicate. This conclusion was further validated by fluorescence dilution experiments involving the fluorescent peptidoglycan precursor D-amino acid 7-hydroxycoumarincarbonylamino-D-alanine (HADA) [33]. Fluorescent bacteria could be detected in LAMP1-labelled vacuoles when macrophages were infected for 6 hours with bacteria grown with HADA (Figure 4*B*). This suggests that new cell wall synthesis, and hence bacterial cell division, did not occur, consistent with the absence of bacterial replication. In contrast, HADA-labelled bacteria growing in culture medium lost blue fluorescence by 3 hours, in contrast to the bacteria treated with the bacteriostatic antibiotic chloramphenicol (Figure 4*C*), which retained fluorescence. Combined, these experiments confirm that 104107 remains viable and persists in human macrophages without replication.

### Intracellular Survival Is Common in Clinical Isolates of Several *Enterobacter* Species

**Figure 5.**
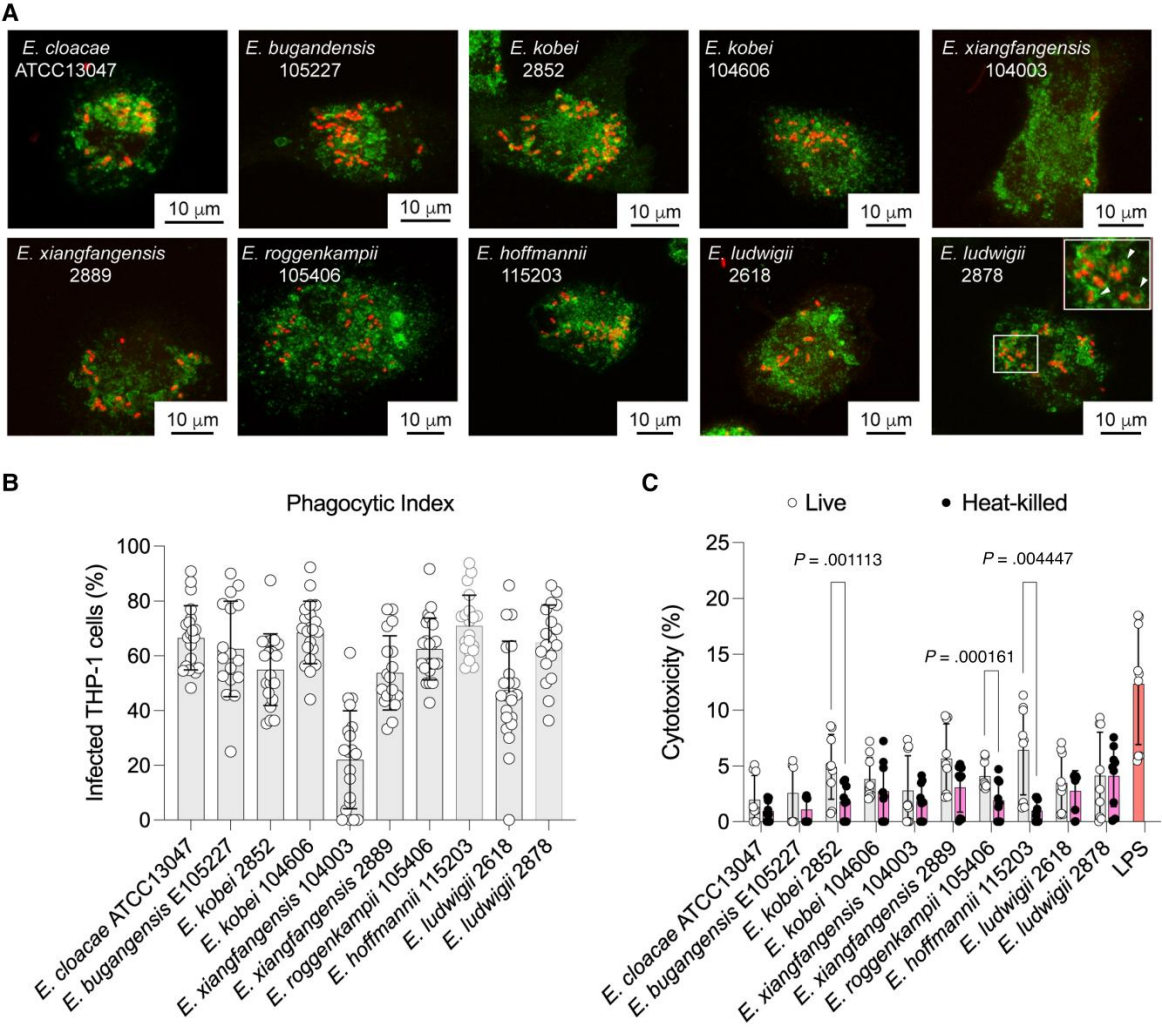
*Enterobacter* clinical isolates traffic to LAMP1-positive compartments in THP-1 macrophages causing minimal cytotoxicity. *A*, THP-1 macrophages infected with live *Enterobacter* isolates (all expressing red fluorescent mCherry) at 3.5 hours postinfection colocalize with LAMP1. Images taken at ×63 magnification on Leica SP8 confocal microscope. MOI = 120. *B*, The percentage of infected macrophages for each isolate was assessed by counting an average of 40 fields of view. *C*, Infected macrophages with live and heat-killed *Enterobacter* isolates display minimal cytotoxicity at 5 hours postinfection, which was measured by lactate dehydrogenase release. Abbreviations: LAMP1, lysosome-associate membrane protein-1; LPS, lipopolysaccharide; MOI, multiplicity of infection. Error bars in panels *B* and *C* denote standard deviation.

Next, we investigated whether the observations with *E. bugandensis* 104107's intracellular survival could be extended to other *Enterobacter* species. We performed THP-1 infections using mCherry-fluorescent derivatives of antimicrobial-resistant *Enterobacter* clinical isolates, which were characterized in a previous study [24]. All these isolates colocalized with LAMP1-positive compartments at 3.5 hpi (Figure 5*A*), and 9 of them infected approximately 60% of macrophages (Figure 5*B*). We conclude that *Enterobacter* bacteria from different species traffic into late phagolysosomes, including the ATCC13047 *E. cloacae* type strain. Notably, all the infecting bacteria induced low levels of macrophage cytotoxicity (ranging from 2% to 7%) in comparison with the 15% cytotoxicity of LPS-treated macrophages (Figure 5*C*), and despite some minor differences in toxicity in some strains when infections with live versus heat-killed bacteria were compared. The intracellular survival of the clinical isolates also did not correlate with their level of colistin/polymyxin resistance, their LPS lipid A structures (Supplementary Figure 7 and Supplementary Table 2), or their levels of relative virulence in the *G. mellonella* infection model (Table 1 and Supplementary Figure 8). Together, these experiments demonstrate that *Enterobacter* clinical isolates survive intracellularly in human macrophages without inducing high levels of cytotoxicity.

**Table 1.** Characteristics of the *Enterobacter* Isolates Comparing Clinical Source, Colistin Resistance Data, Induced Lipid A Modifications, and *Galleria mellonella* Pathogenicity

Characteristic	*Enterobacter* Species									
	*E. cloacae* ATCC13047	*E. bugandensis* E105227	*E. kobei* E2852	*E. kobei* E104606	*E. xiangfangensis* E104003	*E. xiangfangensis* E2889	*E. roggenkampii* E105406	*E. hoffmannii* E115203	*E. ludwigii* E2618	*E. ludwigii* E2878
Clinical score	Spinal fluid	Respiratory secretions	Blood	Respiratory secretions	Respiratory secretions	Blood	Respiratory secretions	Respiratory secretions	Blood	Blood
Colistin resistance^a^	R	R	R	R	S	S	S	S	S	S
Induced lipid A modifications^b^	+	+	+	+	−	−	+	±	−	±
*Galleria mellonella* survival, %^c^	≥ 83	≥ 83	≥ 83	≥ 83	77	≥ 83	40	73	≥ 83	≥ 83

Abbreviations: R, resistant; S, susceptible.

^a^Published in Mushtaq et al [24].

^b^Polymyxin-induced lipid A modifications (see data in Supplementary Figure 7).

^c^Pathogenicity in the *Galleria mellonella* infection model (see data in Supplementary Figure 8).

### Intracellular Survival of *Enterobacter* Isolates Occurs in the Absence of Significant Cytotoxicity and Pyroptosis

**Figure 6.**
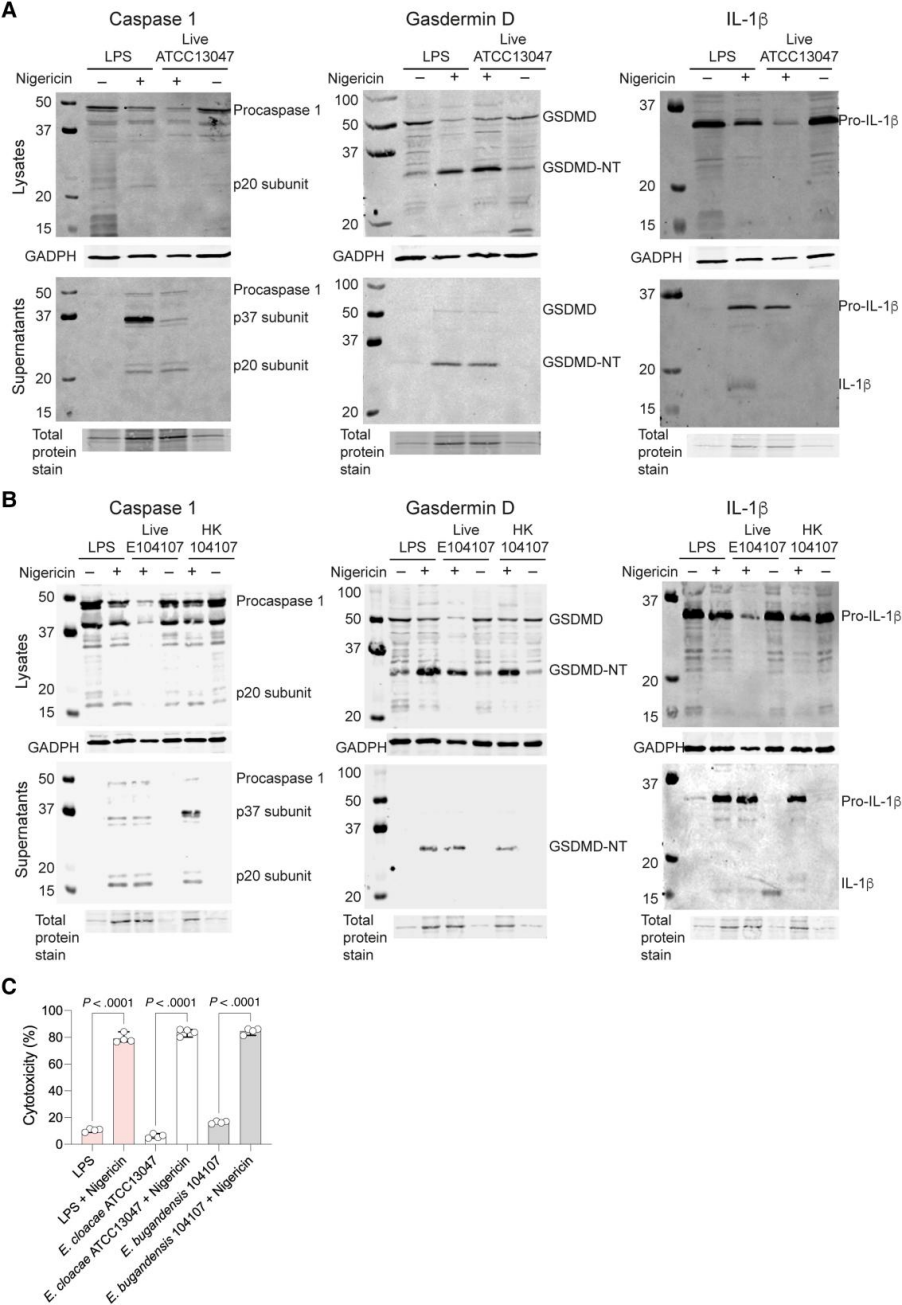
Macrophage responses to *Enterobacter cloacae* ATCC13047 and *Enterobacter bugandensis* 104107 infections. *A* and *B*, Western blots showing full-length and cleaved caspase-1, GSDMD, and IL-1β in lysates and supernatants of live *E. cloacae* ATCC13047-infected THP-1 macrophages (*A*), and live and heat-killed *E. bugandensis* 104107-infected THP-1 cells (*B*). Both sets of experiments were done in triplicate using cells at 5 hours postinfection. MOI = 40. For inflammasome priming and pyroptosis controls, uninfected macrophages were treated with 1 μg/mL LPS and LPS plus 20 μM nigericin, respectively. *C*, Percent cytotoxicity from lactate dehydrogenase assays in culture supernatants of infected macrophages treated and not treated with nigericin, and the LPS and LPS plus nigericin controls at 5 hours postinfection. Error bars indicate standard deviation. Abbreviations: GADPH, glyceraldehyde 3-phosphate dehydrogenase; GSDMD, gasdermin D; GSDMD-NT, GSDMD N-terminal domain; HK, heat-killed; IL-1β, interleukin 1β; LPS, lipopolysaccharide; MOI, multiplicity of infection.

A common outcome of bacterial infection in macrophages is a proinflammatory form of programmed cell death known as pyroptosis [34]. Typically, pyroptosis involves the activation and self-cleavage of procaspase-1, which in turns cleaves GSDMD and pro-IL-1β. The N-terminal domain of GSDMD (GSDMD-NT) oligomerizes and forms a pore in the cell membrane leading to the escape of mature IL-1β and cell lysis [35]. To investigate pyroptosis during *Enterobacter* infection, THP-1 macrophages were infected with live *E. cloacae* ATCC13047 and *E. bugandensis* 104107; western blotting was used to assess the levels of caspase-1 and GSDMD cleavage, and IL-1β secretion in cell lysates and supernatants at 5 hpi. This time point was selected to ensure all intracellular bacteria had completed their trafficking into phagolysosomes. As controls, macrophages were treated with LPS only, LPS plus nigericin (positive control for pyroptosis via the activation of the NLRP3 inflammasome [36]), and live or heat-killed bacteria also in the presence of nigericin. In the experiments with ATCC13047 (Figure 6*A*), the active p20 caspase-1 form was clearly detected in supernatants of nigericin-treated macrophages. The corresponding cell lysates of these samples revealed 2-fold less amount of procaspase-1 (approximately 45 kDa), as determined by densitometry, than macrophages exposed to LPS only or infected with live bacteria. Blots of cell lysates probed for GSDMD revealed the presence of the approximately 31 kDa GSDMD-NT in all cases. However, the amount of this product was 4-fold higher in nigericin-treated cells (Figure 6*A*). GSDMD-NT, which upon GSDMD cleavage oligomerizes and inserts in the cell membrane [37], was also detected in cell supernatants of nigericin-treated cells, suggesting that cell lysis had occurred in these samples. The IL-1β blots revealed a similar pattern showing that the lysates of nigericin-treated macrophages exposed to LPS or infected with live ATCC13047 contained 2-fold and 10-fold less pro-IL-1β, respectively, than the not nigericin-treated macrophages (Figure 6*A*). Both IL-1β and pro-IL-1β were also detected in the supernatants of nigericin-treated cells. Together, the data suggest that cell lysis due to pyroptosis occurs only in the nigericin-treated cells but not in cells infected with live ATCC13047 bacteria.

Similar results were recapitulated using *E. bugandensis* 104107 (Figure 6*B*). In this case, we also included macrophages incubated with live and heat-killed bacteria, both treated and not treated with nigericin. Densitometric analyses indicated similar patterns as shown with *E. cloacae* ATCC13047 infections. The only exception was the detection of IL-1β in the supernatant of not nigericin-treated macrophages infected with live 104107 (Figure 6*B*). Because p20 caspase-1 and GSDMD-NT were not detected, we cannot conclude that 104107 induced pyroptosis, suggesting that the secretion of IL-1β could be due to GSDMD-independent mechanisms [38]. However, this observation was unique to this strain because processed IL-1β was not detected in supernatants from macrophages infected with ATCC13047 (Figure 6*A*) and *E. hoffmannii* 115203 (Supplementary Figure 9). Moreover, detection of lactate dehydrogenase in the supernatants of macrophages infected with live ATCC13047 or 104107, as a proxy for macrophage cell lysis, showed very low levels of cell lysis (≤ 15%) compared with approximately 80% lysis of the macrophages treated with LPS and nigericin (Figure 6*C*). These results demonstrate that macrophages infected with clinical isolates of 3 different *Enterobacter* species do not undergo pyroptosis, supporting the notion that engulfed *Enterobacter* species survive intracellularly in macrophages without inducing significant cytotoxicity.

## DISCUSSION

This study shows that clinical isolates of different *Enterobacter* species survive in human macrophages. Survival occurs in bacteria-containing vacuoles resembling late phagolysosomes that delay acidification, suggesting the bacteria reside in a modified vacuole that delays acidification. Two key features separate *Enterobacter* from other known facultative intracellular bacteria. First, measuring the intracellular bacterial load, or examining the dynamics of wall synthesis in the intracellular bacteria, show that engulfed bacteria do not replicate. Intracellular bacteria remain intact and viable because they express the endogenous mCherry red-fluorescent protein encoded by a plasmid at all time points investigated extending up to 44 hours in HMDMs. Moreover, while bacteria can be recovered at 24 hpi by enumeration of CFUs, no bacteria were cultured in the medium containing kanamycin, an antibiotic that cannot cross eukaryotic cell membranes. These observations imply that *Enterobacter* isolates hide within macrophages as viable, nonreplicating bacteria, joining a long list of bacterial pathogens known to persist in macrophages [39].

We posit that the ability of *Enterobacter* isolates to survive in macrophages provides nonreplicating bacteria with a new strategy to escape antibiotics. This may be clinically relevant, because nonreplicating intracellular *Enterobacter* may escape the action of antibiotics that can penetrate the cell membrane of eukaryotic cells, such as carbapenems, tetracyclines, and fluroquinolones. Survival in murine macrophages can further select for resistant subpopulations of *E. cloacae* against antimicrobial peptides and colistin [40]. Alternatively, infected macrophages could act as Trojan horses to disseminate *Enterobacter*. This idea is reinforced by a study demonstrating the association of *E. hormaechei* with atheromatous tissues and its isolation by cocultivation of plaque tissue homogenates with THP-1 macrophages [17]. Also, certain clinical strains of *E. hormaechei* associated with neonatal sepsis were shown to persist in U937 human macrophages and invade rat brain capillary endothelial cells [16]. However, because our observations are limited to blood-derived monocytic macrophages, it would be premature to extrapolate them to tissue-resident macrophages, which are exposed to different environments and can behave differently [41].

The second salient and surprising feature of *Enterobacter*-macrophage infection is that prolonged bacterial intracellular survival results in minimal macrophage cytotoxicity, suggesting low pathogenicity of *Enterobacter* species. Persistence depends on how well the pathogen can survive in the host, and the balance between the cost of infection versus the costs and effectiveness of the immune response against the infection [42]. Our results agree with the notion that while the magnitude of pathogenicity drives virulence, it does not necessarily increase the rate of host clearance, allowing for persisting bacterial populations [42]. Indeed, we show that survival in macrophages does not correlate with the pathogenicity of our clinical isolates in the *G. mellonella* infection model. Moreover, survival cannot be explained by resistance to antimicrobial peptides because both colistin-resistant and colicin-sensitive isolates can survive in macrophages, or with the lipid A modifications associated with resistance to cationic antimicrobial peptides. Absence of correlation of intracellular survival and colistin resistance further suggests that intracellular bacteria may be able to overcome antimicrobial peptides.

The intracellular *Enterobacter* isolates neither cause significant cell lysis nor pyroptosis, suggesting that intracellular *Enterobacter* could manipulate cell death pathways. In primary human monocytic macrophages, differentiation into proinflammatory or anti-inflammatory types using GM-CSF or macrophage colony-stimulating factor (M-CSF), respectively, do not affect the intracellular fate of *Enterobacter*. While the mechanism of intracellular survival in macrophages without an overt proinflammatory response remains to be elucidated, it may be possible that macrophages can restrict *Enterobacter* replication by targeting key bacterial metabolic pathways, as reported in antibiotic-resistant intracellular *Salmonella enterica* [43]. Alternatively, intracellular *Enterobacter* may contribute to remodeling the macrophages’ cell metabolism. Metabolic reprogramming affecting mitochondrial metabolism and antagonizing programmed cell death by apoptosis to facilitate bacterial persistence in bladder cells has been reported in uropathogenic *Escherichia coli* [44]. *Enterobacter* species form intraepithelial communities in bladder cells, which might provide a reservoir for symptomatic and asymptomatic urinary infections [19]. Metabolic reprogramming in macrophages infected with *Klebsiella pneumoniae*, a pathogen closely related to *Enterobacter,* has been shown to modulate cell death pathways in a bacterial type VI secretion system (T6SS)-dependent manner [45]. A screen for in vivo fitness-associated genes in *E. cloacae* ATCC13047 upon infection in *G. mellonella* discovered several metabolic genes, genes encoding transcriptional regulators, and surface protein- and T6SS-encoding genes [46]. However, it is unclear if T6SS effectors play any direct role in survival because *E. cloacae* does not appear to require T6SS for invasion and proliferation within host cells [18].

## CONCLUSION

This is the first study examining how clinical isolates of various *Enterobacter* species can survive intracellularly in human macrophages. All investigated isolates display multidrug antimicrobial resistance, including some with colistin resistance, and they survive intracellularly in human macrophages. These results underscore the capacity of antimicrobial-resistant *Enterobacter* clinical isolates, traditionally viewed as extracellular bacteria, to hide in macrophages without significantly alerting the innate immune system. We posit our observations have clinical implications because intracellular survival of nonreplicating *Enterobacter* bacterial cells may further complicate the treatment of *Enterobacter* infections and explain treatment failures even in patients infected with antibiotic-susceptible strains [47, 48]. These findings open a door to the development of host-directed therapeutics to enhance bacterial clearance by macrophage-mediated killing [49, 50].

## Supplementary Material

jiaf099_Supplementary_Data

## Supplementary Data

Supplementary materials are available at *The Journal of Infectious Diseases* online (http://jid.oxfordjournals.org/). Supplementary materials consist of data provided by the author that are published to benefit the reader. The posted materials are not copyedited. The contents of all supplementary data are the sole responsibility of the authors. Questions or messages regarding errors should be addressed to the author.
